# CARAMBA: a first-in-human clinical trial with SLAMF7 CAR-T cells prepared by virus-free *Sleeping Beauty* gene transfer to treat multiple myeloma

**DOI:** 10.1038/s41434-021-00254-w

**Published:** 2021-04-13

**Authors:** Sabrina Prommersberger, Michael Reiser, Julia Beckmann, Sophia Danhof, Maximilian Amberger, Patricia Quade-Lyssy, Hermann Einsele, Michael Hudecek, Halvard Bonig, Zoltán Ivics

**Affiliations:** 1 Department of Internal Medicine II, University Hospital of Würzburg, Würzburg, Germany; 2 Institute for Transfusion Medicine and Immunohematology, Goethe University, Frankfurt, Germany; 3 German Red Cross Blood Service BaWüHe, Institute Frankfurt, Frankfurt, Germany; 4 Division of Medical Biotechnology, Paul Ehrlich Institute, Langen, Germany; 5 Department of Medicine/Hematology, University of Washington, Seattle, WA USA

**Keywords:** Myeloma, Immunotherapy

## Abstract

Clinical development of chimeric antigen receptor (CAR)-T-cell therapy has been enabled by advances in synthetic biology, genetic engineering, clinical-grade manufacturing, and complex logistics to distribute the drug product to treatment sites. A key ambition of the CARAMBA project is to provide clinical proof-of-concept for virus-free CAR gene transfer using advanced *Sleeping Beauty* (SB) transposon technology. SB transposition in CAR-T engineering is attractive due to the high rate of stable CAR gene transfer enabled by optimized hyperactive SB100X transposase and transposon combinations, encoded by mRNA and minicircle DNA, respectively, as preferred vector embodiments. This approach bears the potential to facilitate and expedite vector procurement, CAR-T manufacturing and distribution, and the promise to provide a safe, effective, and economically sustainable treatment. As an exemplary and novel target for SB-based CAR-T cells, the CARAMBA consortium has selected the SLAMF7 antigen in multiple myeloma. SLAMF7 CAR-T cells confer potent and consistent anti-myeloma activity in preclinical assays in vitro and in vivo. The CARAMBA clinical trial (Phase-I/IIA; EudraCT: 2019-001264-30) investigates the feasibility, safety, and anti-myeloma efficacy of autologous SLAMF7 CAR-T cells. CARAMBA is the first clinical trial with virus-free CAR-T cells in Europe, and the first clinical trial that uses advanced SB technology worldwide.

## CAR-T-cell immunotherapy is a breakthrough in hematology

Adoptive immunotherapy with gene-engineered chimeric antigen receptor (CAR)-T cells is a major breakthrough and success story for cell and gene therapy. CAR-T-cell therapy targeting the CD19 antigen is an approved treatment for acute lymphoblastic leukemia (ALL), non-Hodgkin lymphoma (NHL), and mantle cell lymphoma, and CAR-T-cell products targeting several alternative antigens are under investigation in hematology and oncology.

**Fig. 1:**
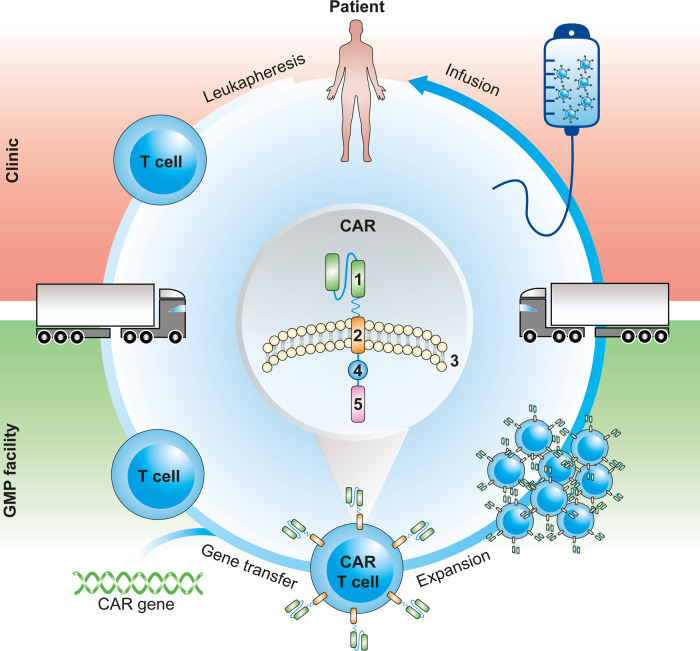
CAR-T-cell manufacturing pipeline. Patients undergo leukapheresis in the clinic to extract T cells, which are transported to a GMP facility, where manufacturing takes place. The CAR gene is introduced into the cells’ genome, leading to receptor expression and conversion into a CAR-T cell. CAR-T cells are then expanded in order to reach therapeutically relevant numbers. Finally, product formulation takes place, and the manufactured product is transported back to the clinic to be infused into the patient. CAR composition: antigen binding domain (1), transmembrane domain (2) spanning the cell membrane (3), costimulatory domain (4), and signaling domain (5).

There are currently three CD19 CAR-T-cell products, i.e., Kymriah™ (lentiviral vector-based), Yescarta™ (γ-retroviral vector-based), and Tecartus™ (γ-retroviral vector-based) that have obtained marketing authorization by the FDA and EMA. All three products are autologous, gene-engineered CAR-T cells that have undergone ex vivo gene delivery followed by re-administration to the patient (Fig. 1). The current ex vivo, autologous CAR-T-cell therapy paradigm comprises a labor-, time-, and cost-intensive supply chain of harvesting the patient’s T cells at a leukapheresis center, shipping to a centralized manufacturing facility to perform CAR gene transfer and T-cell expansion, and return shipment of the cryopreserved cell product to the hospital, where the therapy is administered (Fig. 1).

There is a strong interest and need to facilitate the manufacturing and supply of CAR-T-cell therapy in order to ensure that this treatment can indeed be integrated and become “routine” in clinical practice, and in order to ensure appropriate and equal access of patients to this treatment. We and others consider virus-free gene transfer technology as a key leverage to define a sustainable market price by facilitating streamlined and cost-effective manufacturing of genetically engineered therapeutic cells. Therefore, we have focused on applying virus-free *Sleeping Beauty* (SB) gene transfer for use in CAR-T-cell engineering. The CARAMBA project pioneers the most advanced embodiments of this technology in a first-in-human clinical application.

## The CARAMBA project: bringing cutting-edge CAR-T technology to clinical application

The central objective of the CARAMBA project is to conduct a first-in-human clinical trial with SLAMF7-specific CAR-T cells that have been prepared by virus-free SB gene transfer in patients suffering from multiple myeloma (MM). The CARAMBA consortium comprises ten partners from six EU countries. This includes four clinical centers of excellence in myeloma clinical care and research: University Hospital Würzburg (Germany), Ospedale San Raffaele (Italy), Universidad de Navarra (Spain), and the Centre Hospitalier Regional et Universitaire de Lille (France). Further project partners include the patient organization Myeloma Patients Europe, the Good Manufacturing Practice (GMP) manufacturer DRK-Blutspendedienst Baden-Württemberg-Hessen (BSD-BRK, Germany), the Paul Ehrlich Institute—Federal Institute for Vaccines and Biomedicines (Germany), the biotech companies NBE-Therapeutics, based in Switzerland, and T-CURX, based in Germany, as well as the French project management provider ARTTIC S.A.S. The European Commission selected the CARAMBA project from a large number of highly competitive project proposals in a call for “Innovative treatments for rare diseases” in the Horizon 2020 Research and Innovation program. The kick-off for the CARAMBA project was in January 2018 and the project duration has been scheduled for a 52-month period.

## Key technology in CARAMBA: virus-free gene transfer using the *Sleeping Beauty* transposon system

**Fig. 2:**
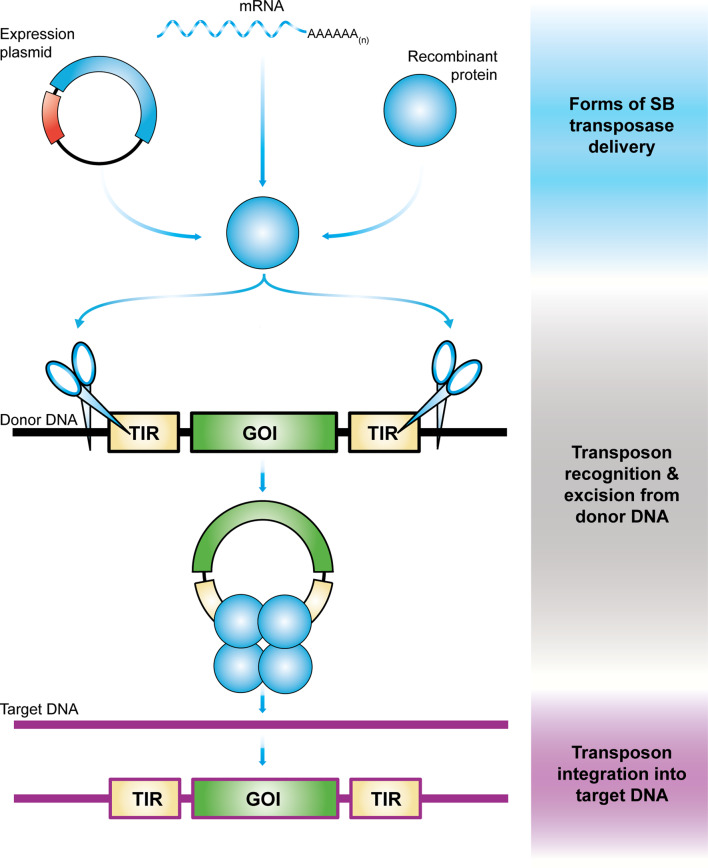
Schematic overview of gene delivery with *Sleeping Beauty* transposition. The SB transposase is introduced into a cell in form of DNA (expression plasmid), mRNA, or recombinant protein along with donor DNA in which the transposon to be mobilized is located. After binding within the terminal inverted repeats of the transposon (TIRs, yellow rectangles) flanking a gene of interest (GOI, green rectangle), SB transposase (blue circles) performs the excision of the transposon from the donor DNA (black strand) and integrates it into a site in the genomic target DNA (purple strand).

DNA transposons are genetic elements with the ability to change their positions within the genome [1]. In nature, these elements exist as mobile (“jumping”) units of DNA containing a transposase gene flanked by terminal inverted repeats (TIRs) that carry transposase binding sites. Importantly, it is possible to separate the two functional components of the transposon (the TIRs and the transposase) in the form of bi-component vector systems (reviewed in [2, 3]). Transposon-based vectors enable incorporation of virtually any DNA sequence of interest between the transposon TIRs and mobilization by *trans*-supplementing the transposase (Fig. 2). In the transposition process, the transposase enzyme mediates the excision of the element from the donor vector, followed by integration of the transposon into a chromosomal locus (Fig. 2). This feature uniquely positions transposons as non-viral gene delivery systems capable of efficient genomic integration that can be used as tools for versatile applications in genetic engineering, including gene therapy (reviewed in [3]).

Based on ancient, inactive transposon sequences isolated from fish genomes, an active transposon was reconstructed, and named SB after the Grimm brothers’ famous fairy tale [4]. SB was the first transposon ever shown capable of efficient transposition in vertebrate cells, thereby enabling new avenues for genetic engineering, including gene therapy (reviewed in [2, 3, 5–14]). The advantage of SB transposon-based gene delivery is that it combines the favorable features of viral vectors with those of naked DNA molecules, namely, (i) permanent genomic insertion of transgene cassettes can lead to sustained and efficient transgene expression [7], (ii) in contrast to non-integrating viral vectors whose repeated in vivo administration can provoke immune responses against vector-encoded proteins (reviewed in [15]), only a single administration of SB vectors is required resulting in diminished immunogenicity in vivo [16], (iii) as opposed to adeno-associated virus vectors [17, 18] and to retrovirus vectors [19, 20], SB vectors have no strict limitation with respect to the size of genetic cargo [21, 22], (iv) superior biosafety profile [10, 23–27], and (v) in contrast to viral vectors, transposon vectors can be maintained and propagated as plasmid DNA, which makes them simple and inexpensive to manufacture [12], an important consideration for implementation and scale-up in clinical practice.

## Therapeutic gene delivery with the *Sleeping Beauty* transposon system

SB transposition-based non-viral gene delivery has an outstanding potential to provide innovative and potentially curative treatments for an array of monogenetic disorders (reviewed in [5–9, 11–14, 28–33]). To name only some, candidate diseases include inborn errors such as tyrosinemia type I, the mucopolysaccharidoses or lipoprotein receptor deficiency, lung diseases like cystic fibrosis, familial pulmonary fibrosis, primary pulmonary hypertension, clotting disorders like Hemophilia A and B, globinopathies like sickle cell disease, dermatological disorders like various forms of epidermolysis bullosa (caused by mutations in certain laminin or collagen genes), muscular dystrophy syndromes, and age-related macular degeneration. Moreover, cancer is considered a therapeutic target, either through direct targeting of tumor cells or indirectly via adoptive immunotherapy approaches (most recently reviewed in [14]).

SB successfully entered the clinical stage in 2011 with two clinical trials as the first non-viral vector being used to generate CD19-specific CAR-T cells for adjuvant immunotherapy targeting minimal residual disease of NHL and ALL after autologous (*n* = 7, ClinicalTrials.gov Identifier NCT00968760) or allogeneic (*n* = 19, ClinicalTrials.gov Identifier NCT01497184) hematopoietic stem cell transplantation (HSCT) [34]. Following autologous HSCT, the 30-month progression-free and overall survivals were 83% and 100%, respectively. After allogeneic HSCT, the respective 12-month rates were 53% and 63%. No acute or late toxicities and no exacerbation of graft-versus-host disease (GvHD) (in the allo-setting) were observed. These pilot studies established safety and illustrated the potential to use SB in CAR-T engineering. In addition, it has been recently reported [35] that allogeneic, donor-derived CD19 CAR cytokine induced killer (CIK) cells engineered with the SB system demonstrated high expansion, low toxicity, and complete remission in patients with relapsed and refractory ALL in a Phase-I/II trial (NCT03389035). CIK cells display an attractive safety profile with minimal occurrence of GvHD after allogeneic cell transplantation; thus, this study provides evidence that donor-derived cells, engineered with the SB transposon, is a safe and valid therapeutic option for ALL patients following allo-HSCT, even if transplanted from mismatched donors. There are currently a total of 14 active clinical trials in gene therapy making use of SB gene transfer technology with additional ones in the planning [14].

The typical setup for delivery of the SB transposon system into cells is supplying the two components of the vector system as conventional plasmids (Fig. 2). However, the use of naked plasmid DNA is associated with several issues that hamper its implementation for gene therapy. First, the efficiency of plasmid DNA delivery into primary human cells by electroporation or by other DNA transfection technologies is generally low [36, 37]. Indeed, the pilot CAR-T clinical applications based on SB gene transfer relied on the use of plasmid vectors supplying transposon and transposase, thereby limiting the levels of stable gene transfer and consequently mandating a long ex vivo culture to obtain the required dose of CAR-T cells to treat patients [34]. Second, transfer of naked plasmid DNA compromises cell viability by a dose-dependent cytotoxicity induced by a type I interferon (IFN) response in human T cells that is independent of TLR 4 or TLR 9 stimulation [38], suggesting that plasmid DNA is recognized by intracellular DNA sensors of the innate immune system. Finally, the presence of an antibiotic resistance gene typically present in plasmid vectors raises safety concerns in the context of gene therapy due to the potential for horizontal gene transfer. Recent advances in SB vector technology have demonstrated that both the efficiency and safety of SB gene delivery can be addressed by the use of minicircle (MC) vectors to encode the transposon and either mRNA synthesized in in vitro transcription reactions or recombinant protein to encode the transposase [38–41] (Fig. 2).

MCs are supercoiled, minimalistic expression cassettes developed for application in non-viral gene delivery. They are derived from their parental plasmids via an intramolecular recombination process, during which the majority of bacterial backbone sequences is depleted from the vector [42]. MC vectors are therefore significantly reduced in size. We consider at least three advantages associated with MC vectors in the context of SB transposon-based gene delivery. First, MCs are superior over plasmids in terms of overall gene transfer efficiency because, due to their smaller size, they cross cellular membranes (the cell membrane and the nuclear membrane) more efficiently than plasmids [43, 44]. Second, the relatively short, ~200-bp distance between the transposon TIRs in MC-based transposon vectors likely aid the transposition reaction [45]. Third, the absence of bacterial plasmid backbone elements in therapeutic vectors is highly relevant in clinical applications, because antibiotic resistance genes included in a therapeutic cell product may raise safety concerns.

The use of in vitro transcribed mRNA to encode the transposase offers additional advantages for therapeutic cell engineering. For example, electroporation of primary human cells, including hematopoietic stem and progenitor cells and T cells, with mRNA was shown to cause significantly reduced cellular toxicity as compared to nucleofection with plasmid DNA [39, 40, 46]. This is likely explained by the inability of RNA to provoke a cellular IFN response in transfected T cells [38]. Second, mRNA bypasses the need for transcription and therefore is, upon transfection, immediately available for protein translation in the cytoplasm. Finally, the implementation of an mRNA source for transient delivery of the SB transposase increases the biosafety of this approach, as mRNA does not bear the risk of chromosomal integration, thereby alleviating a potential risk of genomic instability due to prolonged and uncontrollable transposase expression resulting in continuous remobilization of the already integrated SB transposon.

Implementation of the MC technology in conjunction with synthetic mRNA technologies has recently been shown to enable superior stable gene transfer efficiencies in human T cells for advanced CAR-T-cell engineering with SB transposon vectors [39, 47]. Thus, the CARAMBA consortium has settled to apply a unique combination of mRNA encoding the SB100X hyperactive transposase [37] and an MC vector carrying an SB transposon equipped with a SLAMF7 CAR.

## From bench to bedside: GMP manufacturing of SLAMF7 CAR-T cells

### Strategic setup and approach

**Fig. 3:**
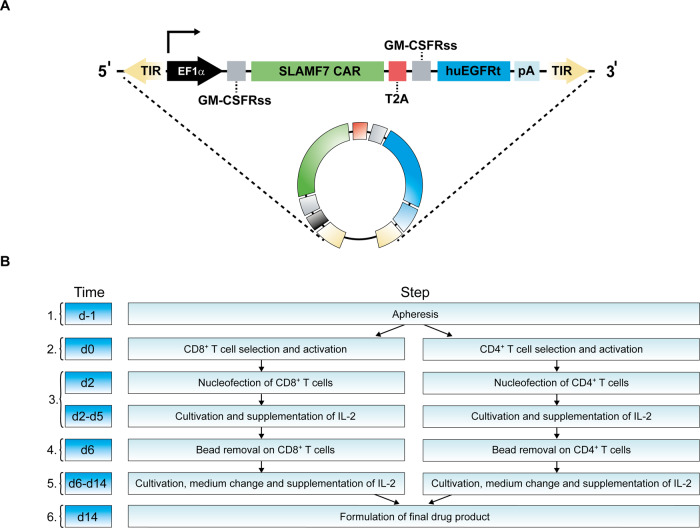
Schematic of the CARAMBA CAR construct and manufacturing process. **A** Schematic of the transposon construct: an EF1α promoter drives expression (arrow) of the SLAMF7 CAR (modeled after the medicinal antibody elotuzumab) and, by means of a T2A element, the human truncated EGFR (huEGFRt). GMCSFRss: GM-CSF receptor-α chain signal sequences. **B** Flowchart of CARAMBA IMP manufacturing. Manufacturing steps are (1) apheresis, (2) immunomagnetic selection and CD3/CD28 activation, (3) nucleofection with SB100X transposase mRNA and SLAMF7 CAR-tEGFR transposon, (4) CD3/CD28-bead removal and transfer to G-Rex culture flasks, (5) half-medium change, and (6) harvest and formulation.

To facilitate the first-in-human CARAMBA trial with autologous T cells targeting the SLAMF7 antigen in refractory MM, an entirely novel manufacturing process was established, which leverages on our preclinical work [48]. The resulting process (Fig. 3) follows the stringent framework of GMP, as laid out by national and European laws and guidelines. The CARAMBA manufacturing process is accompanied by numerous in-process controls (IPCs), to mitigate risks and an extensive panel of formally validated quality control assays, both as pre-defined quality specifications and as “for information only” ancillary tests, which ascertains the pharmaceutical quality of the CARAMBA product (Table 1). Process development followed the usual sequence of technology transfer of the preclinical process from the research laboratory, identification and qualification of suitable starting materials and raw materials, definition of specifications, and development of the necessary assays including formal assay validation, process upscaling, and process validation. A manufacturing authorization for the CARAMBA IMP was obtained, concurrent to the protocol review and approval by the competent regulatory national agencies.

**Table 1:** Quality control assays addressing identity, potency, and safety to ascertain the pharmaceutical quality of the SLAMF7 CAR-T-cell product.

	Method and matrix/time point
*Identity/potency*	
T-cell frequency among CD45^+^ cells	Flow cytometry (drug product)
Transduction efficiency (tEGFR^+^ T cells among all T cells)	Flow cytometry (drug product)
Ratio CD4^+^: CD8^+^ T cells	Flow cytometry (drug product)
Viability (among nucleated cells)	Nucleocounter (drug product)
Dose and volume	Nucleocounter; graduated syringe (drug product)
*Safety*	
Hematopoietic stem/progenitor cells (ISHAGE cells among all CD45^+^ cells)	Flow cytometry (drug product)
Residual myeloma cells (CD138^+^/CD38^+^/7-AAD^−^ cells among CD45^+^ cells)	Flow cytometry [day 0 (post-selection) and, if positive on day 0, on day 14 (drug product)]
Microbiology	BacT/Alert [days 0 (post-CD4^+^/CD8^+^ enrichment, in elution buffer), 6 (post-bead removal, in culture medium) and 14 (drug product)]
Mycoplasma	Microsart^®^ ATMP Mycoplasma; Sartorius [Days 12 (cells in culture medium), 14 (drug product)]
Endotoxin	LAL (drug product)
IDMs (HIV, HBV, HCV, Syphilis)	Patient blood at the time of apheresis; serology and NAT
*Ancillary assays*	
Extended immunophenotyping of CAR-T cells; VCN	Flow cytometry; ddPCR (drug product)

### Implementation of SB gene transfer under GMP

The gene transfer system consists of two components, as highlighted above, which are co-transfected by electroporation (Nucleofector, Lonza, Basel, CH). In contrast to previous therapeutic gene transfer [34], the CARAMBA project takes advantage of two recent technological advances addressing both efficacy and safety of SB gene transfer: in vitro transcribed mRNA (BioNTech, Mainz, Germany) encoding the state-of-the-art hyperactive SB100X transposase [37] and an MC vector (Plasmid Factory, Bielefeld, Germany) [39, 49] encoding the SLAMF7 CAR. The application of mRNA as a source of the transposase enables transient expression and rapid loss of transposase in SLAMF7 CAR-T cells [47], which was crucial to regulatory approval of the CARAMBA trial. The CAR cassette (Fig. 3A) was previously described [48]; it is a conventional second-generation CAR with a CD28-derived costimulatory domain and co-expressing a truncated EGFR (tEGFR) cassette [50] through a T2A sequence. tEGFR is recognized by the medicinal antibody cetuximab and serves three purposes: (i) quantification of CAR-transduced cells during manufacturing and formulation, (ii) detection of CAR-transduced cells in patient blood, and (iii) CAR-T-cell depletion with therapeutic doses of cetuximab in case of severe toxicity [50]. The targeting domain of the CAR is derived from the medicinal anti-SLAMF7-antibody elotuzumab the safety and tolerability of which was shown previously [51].

### Process overview

CARAMBA cell manufacturing (Fig. 3B) takes 14 days in total. Starting from an unmobilized leukapheresis targeting ≥5 × 10^9^ total white blood cells (WBC), the leukocytes are split so that both portions will contain approximately similar total numbers of CD4^+^ and CD8^+^ T cells that are henceforth handled separately, albeit identical and in synchrony (Fig. 3B). These two cell batches are enriched either for CD4^+^ or for CD8^+^ T cells using immunomagnetic selection with clinical-grade antibody-microbead complexes and buffers on research-scale Miltenyi magnetic column in an open system (reagents, consumables, and hardware: Miltenyi Biotech, Bergisch-Gladbach, Germany). The cells are processed sequentially, in an A in B clean room environment. IPCs on the apheresis product include sterility (BacT/Alert hardware and bottles, BioMérieux, Nürtingen, Germany), total viable WBC, and T-cell count separated by CD4^+^ and CD8^+^ counts. IPCs after immunomagnetic separation include recovery, purity, enumeration of total viable CD4^+^ or CD8^+^ cells, respectively [FACS Canto II flow cytometry device, Diva software, antibodies and reagents, Becton-Dickinson, Heidelberg, Germany, except self-labeled cetuximab (ImClone, Eli Lilly, Indianapolis, IN, USA), and Alexa Fluor 647 labeling kit (Life Technologies, Bleiswijk, Netherlands)]. Purity typically approaches 100% with excellent viability, but recoveries in the 20–30% range indicate potential for process improvement. T cells are activated for 2 days, separately for CD4^+^ and CD8^+^ T cells, with CD3/CD28 crosslinking reagent (Dynabeads^®^ CD3/CD28 CTS; Gibco – Life Technologies, Oslo, Norway), co-electroporated with SB100X transposase mRNA and SLAMF7 CAR MC, and expanded for another 12 days in G-Rex culture flasks, with a complete medium change due to bead removal on day 6, followed by a half-medium change on day 12. IL-2 is added every 2nd/3rd day. On day 14, cells are harvested and the drug product is formulated at a 1:1 ratio (permissible range: 0.5:1–2:1) of CD4^+^ and CD8^+^ CAR-positive T cells. A cell dose corresponding to the targeted dose range is suspended in saline buffer to a volume of 1 mL/kg body weight. Quality control assays, both specification-describing and ancillary, are performed on the bulk mix of CD4^+^CAR^+^ and CD8^+^CAR^+^ and/or final drug product. The CARAMBA product is infused fresh as soon as possible but not later than 48 h after formulation. At 4–8 °C, the CARAMBA product is stable for at least 72 h, as was formally demonstrated in designated stability exercises.

### Formulation and shipment

A particular challenge is the characteristically wide range of T-cell doses that are administered in the process of a Phase-I study with a dose-finding arm, since the lower doses result in cell concentrations well below the technical level of detection of conventional cell counters and thus prohibiting assessment of cell dose in the final drug product that contains the respective dose—as little as 10,000 cells—in a volume of 1 mL/kg body weight of the recipient. The final infusion bag is overfilled by at least 20%; the overfill is immediately withdrawn for sterility, endotoxin (Endosafe nexgen PTS hardware and consumable, Charles River, Sulzfeld, Germany) and mycoplasma testing (Microsart® ATMP Mycoplasma, Sartorius, Göttingen, Germany) as well as for cryopreservation of retain samples and for ancillary tests. The latter include vector copy number analysis, using digital droplet PCR (QX200, BioRad, Feldkirchen, Germany), and extensive T-cell phenotyping. Availability of residual cells, provided in vitro and in vivo cytotoxicity, is also assessed, using a 4-hour Europium Assay analyzed using a Victor X4 Multilabel plate reader (Perkin Elmer, Rodgau, Germany) or xenografted NSG mice with MM.1S cells (ATCC ref. CRL-2974) as targets, as well as residual SB100X protein by western blot and vector insertion site profile analyses. All of the latter, not specification-defining assays, follow formally established protocols and include relevant controls, but unlike those defining the product specification are not validated according to guidance of the European Pharmacopoeia. The CARAMBA product is released to the patient by a Qualified Person of German Red Cross Blood Service and a sponsor representative.

## The CARAMBA clinical trial: first-in-human application of SLAMF7 CAR-T cells

The CARAMBA clinical trial comprises a Phase-I dose escalation part, and a Phase-IIa dose expansion part, and will recruit up to 38 patients with MM. The CARAMBA clinical trial will recruit at specific sites in Germany, Spain, France, and Italy. There are a number of inclusion and exclusion criteria that patients have to fulfill in order to participate in the CARAMBA clinical trial, e.g., patients must have received at least three prior lines of treatment, including a proteasome inhibitor (such as bortezomib and carfilzomib), an immunomodulatory agent (such as lenalidomide or pomalidomide), a monoclonal antibody (such as daratumumab), and most have undergone high-dose chemotherapy with subsequent autologous HSCT, to be eligible for this procedure. Furthermore, patients must have measurable disease markers of myeloma. Patients who have previously received treatment with elotuzumab, a monoclonal antibody targeting SLAMF7, are eligible to take part in the CARAMBA clinical trial. Because CAR-T-cell therapy can be quite an intensive treatment with potential side effects such as cytokine release syndrome (CRS) and neurotoxicity, patients are also required to have a good performance status, which is measured using the Eastern Cooperative Oncology Group (ECOG) score. Patients must have an ECOG score of less than 2, implying a greater level of fitness, to participate in the trial. Patients must have adequate heart, liver, and kidney function as assessed by an ultrasound and bloodwork prior to determining their eligibility for the CARAMBA trial. Patients who underwent a prior allogeneic HSCT are eligible for the CARAMBA trial if HSCT has taken place at least 12 months prior to the planned day of leukapheresis. Patients with GvHD and taking medications to suppress their immune system or patients with an active infection including HIV, syphilis, hepatitis B, and hepatitis C are not eligible for participation in the CARAMBA trial. The key analysis endpoints in the CARAMBA clinical trial include measures of feasibility of using SLAMF7 CAR-T cells as treatment for myeloma, safety as assessed by the number and severity of adverse events (such as CRS and neurotoxicity), and anti-myeloma efficacy. Also, quality of life and hospital resource utilization in the context of CAR-T-cell therapy will be measured in the CARAMBA clinical trial.

## Perspective: evidence for CAR-T-cell therapy in multiple myeloma

MM is a rare hematologic malignancy of aberrant plasma cells. The conventional treatment of MM comprises chemotherapy and newer agents like proteasome inhibitors; however, over the past decade MM treatment is being redefined by humoral and cellular immunotherapies. CAR-T-cell therapy is the most exciting new development in MM therapy, and there is emerging clinical evidence from the use of CAR-T cells targeting the B cell maturation antigen BCMA. Several BCMA CAR-T-cell products are currently in clinical development, and have accomplished very high rates of responses in patients who had relapsed and were unresponsive (refractory) to all established anti-MM treatments [52]. Overall, BCMA CAR-T-cell therapy has displayed a favorable safety profile, with a low incidence of CRS and neurotoxicity. However, the clinical experience with BCMA CAR-T cells has also exposed several challenges associated with targeting this antigen, and potential mechanisms of relapse or resistance include antigen downregulation or even loss [53]. Several strategies are being pursued to address these challenges, including the use of γ-secretase inhibitors to enhance BCMA molecule density on MM cells and reduce the amount of soluble BCMA in serum [54] and use of CAR products with defined T-cell subset compositions and humanized targeting domains to reduce immunogenicity and promote engraftment and in vivo expansion [55].

**Fig. 4:**
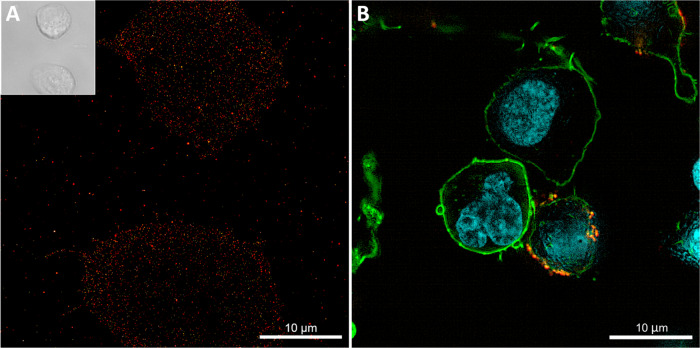
Multiple myeloma cells expressing SLAMF7 and targeting them with CAR-T cells. **A** dSTORM image showing SLAMF7 molecules expressed on MM.1S (ATCC ref. CRL-2974) cells. Cells were stained with anti-SLAMF7 Alexa Fluor 647 antibody. The inset displays a bright field image of the same cells. **B** Confocal microscope image showing the interaction between a SLAMF7 CAR-T cell and a MM.1S target cell. Cells were stained with phalloidin Atto643 for actin (green), with anti-CAR CF568 antibody (orange) and Hoechst 34580 for nuclei (blue).

In addition, there is an ongoing effort to identify and validate additional CAR target antigens in MM, and we have focused on evaluating SLAMF7 as a novel candidate antigen in the CARAMBA trial. Several studies have demonstrated high-level, uniform expression of SLAMF7 on malignant plasma cells; a representative image is displayed in Fig. 4A. Importantly, SLAMF7 expression is retained in MM patients with relapsed/refractory disease, and after intensive prior therapy [48, 56, 57]. In previous work, we have demonstrated that T cells expressing a SLAMF7 CAR (Fig. 4B) are substantially more potent against MM than the anti-SLAMF7 antibody elotuzumab in preclinical models in vitro and in vivo [48] and, therefore, the results of the CARAMBA clinical trial are eagerly awaited.

## Outlook: impact of the CARAMBA project on the CAR-T landscape in Europe

There are hundreds of entries that emerge on ClinicalTrials.gov by using “CAR-T” as a query term. Indeed, clinical trials targeting a wide range of antigens for hematologic and solid tumors are currently under development; thus, it is predicted that the demand for CAR-T-cell therapies will increase in the near future. However, costs revolving around manufacturing of autologous cell products (every single therapeutic cell batch being uniquely suitable only for the patient from whom the cells had been isolated) are expected to become a major bottleneck, potentially limiting the access to these novel drugs in entire geographical regions and societies. In addition, the manufacturing capacities of centralized and highly specialized GMP production facilities will likely be exhausted, especially if CAR-T therapies will become available for more common forms of cancer affecting larger patient populations. Thus, in order to address the significant and foreseeable demand for CAR-T therapies in the near future [58] and to enable broad access to this therapy, manufacturing protocols that are more suitable to reduce production time and costs of the cell product are currently under investigation and, in this regard, the advances made in the CARAMBA project constitute a breakthrough into a new era in CAR-T-cell therapy.

SB holds significant potential to lower the costs associated with manufacturing of gene therapy products such as CAR-T cells, and thus enhancing availability for the general public. Current estimations range around a 90% reduction in vector manufacturing costs on a per patient basis [12], as GMP-grade manufacturing of naked nucleic acids, as required for the SB system, is easily scalable and does not require work-intensive and time-costly additional quality control. SB technology promises several additional advantages over viral gene transfer, namely, lower biosafety level translating to lower infrastructure costs for manufacturing and quality control and high modularity. Recently, some modifications of the SB100X protein have allowed generation of recombinant, soluble SB protein [41], which in the future will allow for even shorter ex vivo cultivation, with the obvious biological and financial benefits.

Separate generation and expansion of CAR-modified CD4^+^ and CD8^+^ T cells allows for optimized formulation of the two at a ratio of approximately 1:1, even though it may sacrifice some expansion potential especially of CD8^+^ T cells due to lack of T cell help, which, however, is easily compensated by higher starting cell numbers. Separation of CD4^+^ and CD8^+^ cells uniquely permits use of different CAR constructs as emerging evidence seems to suggest differential preference for costimulatory domains favoring brisk onset and proliferation in CD8^+^ and longevity in CD4^+^ cells [55, 59, 60], as future studies will have to ascertain for SLAMF7 and MM. The highly modular process established to support the CARAMBA study can easily and inexpensively be adapted to any other CAR- or TCR-T or NK effector cell products, and thus possesses value that goes far beyond CARAMBA and MM.

Given the financially very significant engagement of major pharmaceutical companies in the fields of cell and gene therapy and immuno-oncology with genetically modified (typically autologous) immune effector cells, the question why academia remains active in this highly competitive field is often posed. The amount of public research funding available is already quite limited, size and duration of typical grants fall significantly short of what is required for proper pharmaceutical development, and the probability of failure of any such proposal is outsize. However, for the foreseeable future we anticipate that the majority of innovations in the fields of cell and gene therapy in general and CAR-T-cell therapy in particular will continue to derive from academic institutions until industry develops its own expertise and gets comfortable with the risk and courage that it takes to bring a truly novel cell product from the bench to first clinical application. Ever so often, these ideas will be taken over by industry and developed to marketable medicines, and we predict that some of the innovations in CARAMBA will be among them.
